# Beneficial effect of Indigo Naturalis on acute lung injury induced by influenza A virus

**DOI:** 10.1186/s13020-020-00415-w

**Published:** 2020-12-21

**Authors:** Peng Tu, Rong Tian, Yan Lu, Yunyi Zhang, Haiyan Zhu, Lijun Ling, Hong Li, Daofeng Chen

**Affiliations:** 1 Department of Natural Medicine, School of Pharmacy, Fudan University, No. 826, Zhangheng Road, Shanghai, 201203 People’s Republic of China; 2 Department of Pharmacology, School of Pharmacy, Fudan University, No. 826, Zhangheng Road, Shanghai, 201203 People’s Republic of China; 3 Department of Microbiological and Biochemical Pharmacy, School of Pharmacy, Fudan University, No. 826, Zhangheng Road, Shanghai, 201203 People’s Republic of China

**Keywords:** Influenza, Indigo Naturalis, Acute lung injury, Cytokines, Inflammation

## Abstract

**Background:**

Infections induced by influenza viruses, as well as coronavirus disease 19 (COVID-19) pandemic induced by severe acute respiratory coronavirus 2 (SARS-CoV-2) led to acute lung injury (ALI) and multi organ failure, during which traditional Chinese medicine (TCM) played an important role in treatment of the pandemic. The study aimed to investigate the effect of Indigo Naturalis on ALI induced by influenza A virus (IAV) in mice.

**Method:**

The anti-influenza and anti-inflammatory properties of aqueous extract of Indigo Naturalis (INAE) were evaluated in vitro. BALB/c mice inoculated intranasally with IAV (H1N1) were treated intragastrically with INAE (40, 80 and 160 mg/kg/day) 2 h later for 4 or 7 days. Animal lifespan and mortality were recorded. Expression of high mobility group box-1 protein (HMGB-1) and toll-like receptor 4 (TLR4) were evaluated through immunohistological staining. Inflammatory cytokines were also monitored by ELISA.

**Result:**

INAE inhibited virus replication on Madin-Darby canine kidney (MDCK) cells and decreased nitric oxide (NO) production from lipopolysaccharide (LPS)-stimulated peritoneal macrophages in vitro. The results showed that oral administration of 160 mg/kg of INAE significantly improved the lifespan (*P* < 0.01) and survival rate of IAV infected mice, improved lung injury and lowered viral replication in lung tissue (*P* < 0.01). Treatment with INAE (40, 80 and 160 mg/kg) significantly increased liver weight and liver index (*P* < 0.05), as well as weight and organ index of thymus and spleen at 160 mg/kg (*P* < 0.05). Serum alanine transaminase (ALT) and aspartate aminotransferase (AST) levels were reduced by INAE administration (*P* < 0.05). The expression of HMGB-1 and TLR4 in lung tissue were also suppressed. The increased production of myeloperoxidase (MPO) and methylene dioxyamphetamine (MDA) in lung tissue were inhibited by INAE treatment (*P* < 0.05). Treatment with INAE reduced the high levels of interferon α (IFN-α), interferon β (IFN-β), monocyte chemoattractant protein-1 (MCP-1), regulated upon activation normal T cell expressed and secreted factor (RANTES), interferon induced protein-10 (IP-10), tumor necrosis factor-α (TNF-α), interleukin-6 (IL-6) (*P* < 0.05), with increased production of interferon γ (IFN-γ) and interleukin-10 (IL-10) (*P* < 0.05).

**Conclusion:**

The results showed that INAE alleviated IAV induced ALI in mice. The mechanisms of INAE were associated with its anti-influenza, anti-inflammatory and anti-oxidation properties. Indigo Naturalis might have clinical potential to treat ALI induced by IAV.

## Introduction

Viruses infection has threatened human health. The current outbreak of novel coronavirus disease 19 (COVID-19), caused by severe acute respiratory coronavirus 2 (SARS-CoV-2), has become a big threat to the world [1]. The growing infection is second only to the influenza virus infection occurred in 1918, which killed more than 50 billion people worldwide, and resulted in havoc with military operations during the First World War [2].

Influenza A virus (IAV) is one of the important human pathogens worldwide. IAV infection can cause acute respiratory distress syndrome (ARDS), pneumonia, and lead to high mortality and morbidity [3]. Just like the current coronavirus pandemic which has spread over 200 countries and regions, fast spread of IAV infection may cause epidemic and threat people’s safety and property [4].

IAV is negative strand RNA virus belonging to the family of orthomyxoviridae with a genome consisting of eight single stranded RNA segments of negative polarity [5]. Vaccine, M2 ion channel inhibitors and neuraminidase inhibitors are most used for treatment of IAV infection [6]. However, the lack of timeliness of vaccines and continuous records about drug resistance to influenza virus impetus the demands for new alternative antivirus substance, especially from nature products and traditional Chinese medicine (TCM) [7].

Acute lung injury (ALI) is characterized by severe lung edema and inflammation, and the pathogenesis of ALI involves immune imbalance [8]. Acute lung injury is the most common form of influenza A virus infection, during which the host alveolar epithelial cells are infected and injured [9]. When the host is infected, the virus can induce a series of signaling cascades to their own benefit, such as toll-like receptors (TLRs) and other signaling pathways [10]. As we all know, pathogen-associated molecular pattern (PAMP), as well as damage-associated molecular pattern (DAMP) activate antigen-presenting cells through TLRs to initiate immune responses [11]. DAMPs such as high mobility group box-1 protein (HMGB-1) are released early from the infected host cells in the extracellular medium in response to viral stimuli. Influenza virus infection can increase the expression of TLR4 in lung [12]. HMGB-1 is detected by TLR4 and leads to amplify inflammation and exacerbate tissue injury [13]. The binding of HMGB-1 to TLR4, as well as the recruitment of neutrophils to the infected areas accelerated the production of large amounts of oxidative products and proinflammatory cytokines, and further aggravated lung injury [14]. Influenza virus infection and COVID-19 both result in respiratory system damage and perhaps share similar infection process. Besides pneumonia and acute lung injury, other vital organs injury, such as liver, thymus, spleen, heart or kidneys dysfunction were observed in patient suffered from COVID-19 [15].

Traditional Chinese medicine, the precious treasure of China which stem from antique Chinese culture 2000 years ago, are used to treat diseases in China and southeast Asia. TCM played a significant role in the treatment of COVID-19, bringing new hope for the prevention and control of COVID-19 and influenza virus [16].

Indigo Naturalis, a dark blue powder, mass or granules, is prepared from stems and leaves of *Baphicacanthus cusia* (Nees) Bremek. (Fam. Acanthaceae)*, Polygonum tinctorium* Ait. (Fam. Polygonaceae) *or Isatis indigotica* Fort. (Fam Cruciferae) [17, 18]. Indigo Naturalis (Qingdai) is among the family of the traditional Chinese medicine with heat-clearing and detoxifying capacity. Indigo Naturalis as a folk traditional Chinese medicine is used to treat psoriasis, colitis and upper respiratory system diseases [19]. Jin-mo Shi, the famous doctor of traditional Chinese medicine used Indigo Naturalis to treat parotitis, acute and cholic pharyngitis and armydalitis [20].

Researchers have confirmed the definite effect of Indigo Naturalis on psoriasis and colitis, and the mechanisms of action has also been studied [21]. Indigo Naturalis reduced the expression of cytokines and chemokines and inhibited the proliferation of keratinocytes and endothelial cells to treat psoriasis [22]. However, there are few reports on the effects and mechanisms of Indigo Naturalis on respiratory virus infection. In this experiment, a mouse model of IAV infection was established to study the effects and mechanisms of Indigo Naturalis on IAV-induced ALI.

## Methods

### Reagent

The powdered form of Indigo Naturalis was prepared from the leaves of *B. cusia* (Nees) Bremek and purchased from Shanghai Ley’s Pharmaceutical Co., Ltd., with the place of production of Xianyou County, Fujian province, China. The material was identified by the authors. The voucher specimen of Indigo Naturalis (DFC-LF-201509) was deposited in Department of Natural Medicine, School of Pharmacy, Fudan University.

Ribavirin was purchased from Shanghai Meryer chemical Co., Ltd.. ELISA kits for mouse tumor necrosis factor-α (TNF-α), interleukin-6 (IL-6), monocyte chemoattractant protein-1 (MCP-1), regulated upon activation normal T cell expressed and secreted factor (RANTES), interleukin-10 (IL-10), and interferon induced protein-10 (IP-10) were purchased from Shanghai Boatman Biotechnology Co. Ltd.. ELISA kits for interferon-α (IFN-α), interferon β (IFN-β), interferon γ (IFN-γ), myeloperoxidase (MPO), methylene dioxyamphetamine (MDA) were purchased from Shanghai Beyotime Biotechnology Co., Ltd.. The anti-HMGB-1 and anti-TLR4 antibodies were purchased from Abcam (San Francisco, CA, USA). Alanine transaminase (ALT) and aspartate aminotransferase (AST) were determined using Roche Cobas 6000 c501 Chemistry Analyzer (Roche Diagnostics, Mannheim, Germany) and supporting reagents.

### Herbal extract preparation

The Indigo Naturalis powder (2 kg) were packaged with 4 layers of etamine, and then extracted with boiling water for 1 h with three times. The water extract was concentrated under reduced pressure and lyophilized to produce 82 g of aqueous extract of Indigo Naturalis (INAE).

### Chemical components identification from INAE

Analysis was performed using an UPLC-IT/MS system (Thermo, Finnigan, San Jose, CA, USA). Three milliliter of deionized water was added into 3 mg of powdered INAE, after vortex and sonication, the 1 mL/mg INAE stock solution was filtered through a 0.22 μm filter (Millipore) before injected to the UPLC-IT/MS analysis.

A YMC-Triart C_18_ column (150 × 2.1 mm i.d., 1.9 μm; YMC, Japan) was used for sample preparation, with an injection volume of 5 μL for the constituent separation and analysis on the Dionex Ultimate-3000 UPLC-IT/MS Velos Pro ion-trap mass spectrometer system equipped with electrospray ionization source in positive ion mode. The mobile phases consisted of water (A) and acetonitrile (B) at a flow rate of 0.3 mL/min. The MS parameters were optimized as follows: selections of the target mass range *m/z* of 110 ~ 2000; compound stability 100%; trap drive level 100%; collision energy 1 V; auxiliary gas (N_2_) flow rate of 10 L/min; the capillary temperature of 350^◦^C; dry gas 8 L/min; capillary voltage of 30 V; sheath gas (N_2_) flow rate of 15 L/min; and nebulizer gas 40 psi were made by examination of the full grade scan intensity, stability, and the product ions spectra.

### In vitro antiviral evaluation

The influenza A virus (H1N1 A/FM/1/47) was denoted by vice professor Haiyan Zhu, Department of Microbiological and Biochemical Pharmacy, School of Pharmacy, Fudan University. According to the reference and procedures in our laboratory, the virus was suspended in Dulbecco’s modified Eagle’s medium (DMEM), propagated in lung of mice and stored at − 80 °C. The 50% lethal dose (LD_50_) of virus was determined in mice infected with serial dilutions of virus [23].

Once attaching to host cells, virus propagate in and release virions from infected host cells [24]. In vitro antiviral evaluation was conducted as described with minor modification [25]. Madin-Darby canine kidney (MDCK) cells were cultured in DMEM supplemented with 10% fetal bovine serum (Hyclone, CA, USA), streptomycin and penicillin. Briefly, MDCK cells were cultured in 96-well plates (1 × 10^5^ cells per well) till cells fulfilled 90% percent of the bottom. INAE were diluted and prepared in concentrations of 0, 10, 25, 50, 100, 200 and 400 μg/mL. The antiviral process was investigated based on three ways of action: added the drugs 2 h prior to, incubated the drugs with 100 TCID_50_ influenza A virus, or 2 h after virus infection. The cells with only infection virus were served as virus control. After 3-day’s incubation, 3-(4,5-dimethyl-2-thiazolyl)-2,5-diphenyl-2-H-tetrazolium bromide (MTT) was added into the wells. Following 4 h incubation, the optical density value (OD value) of supernatant was tested. Inhibition rate (%) = [(OD of INAE – mean OD of virus control)/(mean OD of cells control—mean OD of virus control)] × 100%.

### In vitro anti-inflammatory effect

BALB/c mice were administrated with 5% mercaptoethanol acid sodium by intra-peritoneal injection and scarified 4 days later to harvest peritoneal macrophages. Macrophages were suspended with RPMI-1640 culture medium containing 10% fetal bovine serum and antibiotics. The cells were cultured in 96 cell plate (1 × 10^6^ cells per well) for 2 h. The serial dilutions of INAE, lipopolysaccharide (LPS, 1 μg/ml), and dexamethasone (positive control, 10 μM) were added and incubated for 24 h. A sample of cells without LPS was set aside to serve as a control. The supernatant was collected at the end of the incubation. The nitrogen oxide (NO) concentration in the supernatant was calculated by measuring the OD value of supernatant following the instruction of method using Griess reagent [26].

### Animals

Specific pathogen-free male BALB/c mice (14–16 g) were purchased from Shanghai SLAC Laboratory Animal Co., Ltd. [SCXK (Hu) 2012-0002]. The mice were housed in collective cages under a 12 h light/dark room, with free access to food and water. The air temperature was maintained at 22 ± 2 °C with relative humidity of 50 ± 10%. Experiments were carried out according to the guideline for the care of laboratory animals of National Institutes of Health. All study protocols were approved by the animal ethical committee of School of Pharmacy, Fudan University (approval No. 2015-10-SY-CDF-01).

### Survival experiment

Survival experiment is the usual and important experiment to evaluate the effects of compound on reducing the lethality of IAV infection [3]. The survival experiment was conducted as described with minor modification [27]. Mice were randomly divided into six groups (n = 10): normal, model, INAE (40, 80 and 160 mg/kg) and positive control (ribavirin, 100 mg/kg). Mice were anaesthetized by tail intravenous injection of propofol (0.026 mL/10 g) and were intranasal challenged with 6 × LD_50_ IAV in 30 μL of RPMI-1640 medium 2 h before the compound treatment. Normal mice were challenged with 30 μL of RPMI-1640 medium. The mice were treated orally with INAE or ribavirin once daily for 7 days. For comparison, normal group and model group were given 0.5% carboxymethyl cellulose sodium (CMC-Na). All groups were monitored for 14 days after virus infection. Body weight, body temperature, and animal clinical health were monitored daily. Lifespan and mortality rate of mice were calculated.

### Establishment of acute lung injury induced by IAV

In order to study the efficacy and mechanisms of INAE on IAV infection, acute lung injury in mice was induced by IAV infection in a 4-day experiment [8]. The experiment was scheduled by six groups (n = 6): normal, model, INAE (40, 80 and 160 mg/kg) and positive control (ribavirin, 100 mg/kg). Mice were anaesthetized by tail intravenous injection of propofol (0.026 mL/10 g) and then inoculated intranasally with 3 × LD_50_ IAV suspended in 30 μL of RPMI-1640 medium. Normal mice were challenged with 30 μL of RPMI-1640 medium. Treatment was initiated 2 h after virus infection. The mice were treated orally with INAE or ribavirin once daily for 4 days. Mice in normal group and model group were given 0.5% CMC-Na. The mice were sacrificed on day 4 after virus infection. The lung tissues were harvested, weighted and then washed with pre-chilled saline. The superior right lobe was cut and placed in 10% neutral buffered formalin for histopathologic evaluation, and the rest parts were snap frozen at − 80 °C for cytokine detection. To estimate the severity of lung, liver, thymus and spleen, organ indexes were calculated as follows: Organs index = Organ weight (mg) / body weight (g) × 100%. Serum ALT and AST levels were also detected to evaluate the severity of liver injury.

Lung tissues were fixed in 10% neutral buffered formalin. After fixation, the samples were dehydrated and embedded in paraffin. The embedded samples were cut into 5-μm slices and stained with hematoxylin and eosin (H&E). Lesions in lung were observed by optical microscopy. Lung injury were determined through the severity of pneumonia in a blinded fashion [27].

After rehydrated through a graded series of alcohol, the slices were incubated with rabbit antibodies against HMGB-1 (1:200 diluted) and TLR4 (1:250 diluted) at 4 °C overnight. The sections were then incubated with special HRP-conjugated goat anti-rabbit IgG antibody at 37 °C for 30 min. Slides were stained with chromogenic substrate solution diaminobenzidine (DAB) and counterstained with hematoxylin. The expression of HMGB-1 and TLR4 were visualized under a microscope [28]. The expression of HMGB-1 and TLR4 were also assessed by semi-quantitative analysis using ImageJ software.

### Determination of lung virus titer

The frozen lung tissues were thawed and homogenized in phosphate buffered saline (PBS) at a concentration of 100 mg tissue/1 mL PBS as described with minor modification [29]. The supernatant was split into aliquots and stored at -80^◦^C for subsequent use.

MDCK cells were plated at 2 × 10^6^ cells in 96 cell plate till the cells grown to 90% confluent, cells were then infected with serial dilutions of supernatant from lung homogenate and incubated for 2 h at 37 °C with 5% CO_2_. The supernatant was removed and the wells were washed with PBS and incubated with 200 μL DMEM at 37 °C, 5% CO_2_ for 3 days. The cell activity was assessed by MTT assays. The lung virus titer was expressed by the inhibition rate of virus replication [30].

### Anti-oxidant capacity in supernatant of lung homogenate

The anti-oxidation capacity of INAE was tested through the method of ferric ion reducing antioxidant power (FRAP). Levels of MPO and MDA, the peroxide products in lung homogenate of IAV infected mice were also evaluated by detection kits according to the manufacturer’s instructions.

### Assessment of cytokines in supernatant of lung homogenate

Levels of IFN-α, IFN-β, IFN-γ, MCP-1, RANTES, IP-10, TNF-α, IL-6, and IL-10 in lung homogenate of IAV-infected mice were determined with ELISA kits according to the manufacturer’s instructions.

### Statistical analysis

All parameters were recorded for individuals within all groups and statistical computations were performed with GraphPad prism 6 (GraphPad software Inc., San Diego, CA, USA). Data comparison were carried out with one-way ANOVA and expressed as mean ± S.D. (Standard deviation). Post hoc comparisons were performed using Fishers’s PLSD if any significant changes were found. The *P* values less than 0.05 were considered as statistically significant.

## Results

### Chemical components identification from INEA

**Fig. 1:**
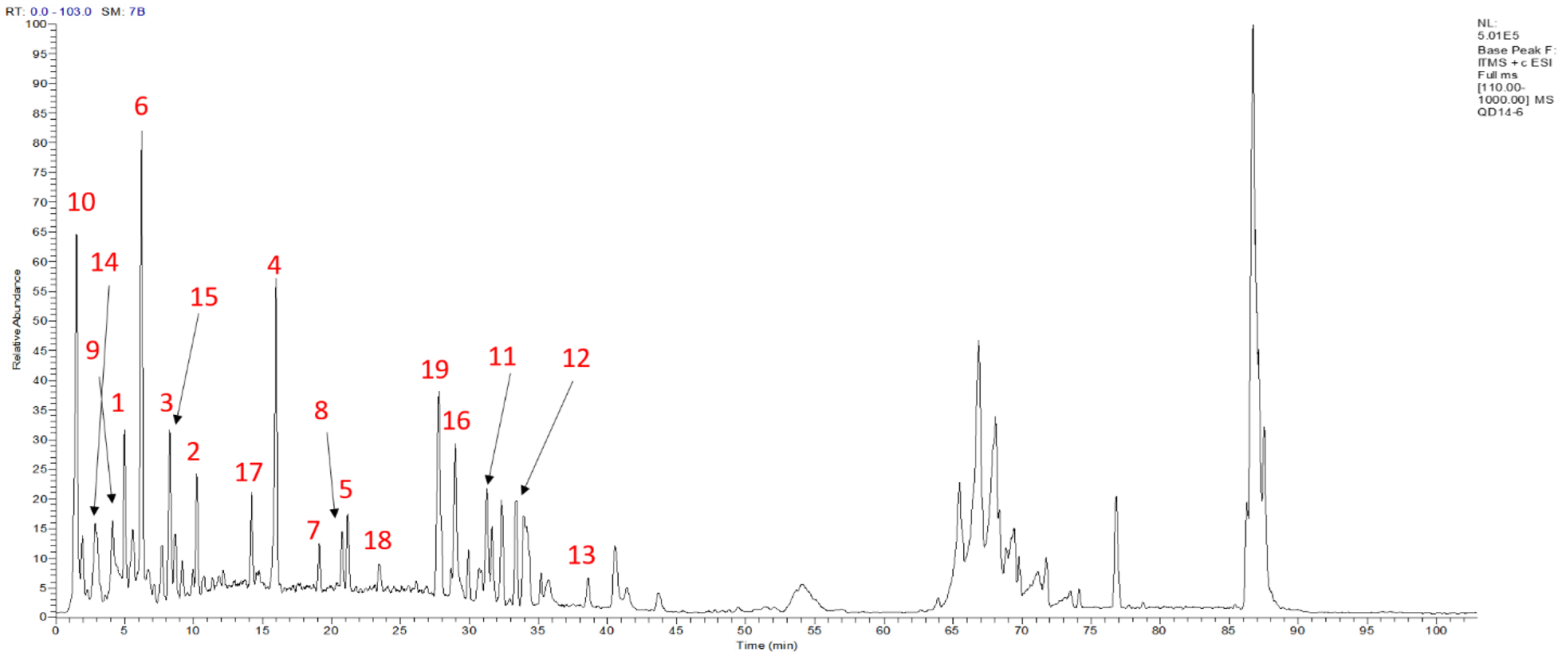
UPLC-ESI-LTQ-MS positive ion mode chromatogram of INAE. The aqueous extract of Indigo Naturalis (INAE) was diluted in deionized water and filtered through a 0.22 μm filter before introduced to the UPLC-ESI-LTQ-MS analysis. A YMC-Triart C_18_ column was used with an injection volume of 5 μL for the constituent separation and analysis in positive ion mode

The chemical components of INAE was identified by UPLC-ESI-LTQ-MS analysis system. Positive mode chromatography was chosen to characterize the chemical constituents for more plentiful chromatographic peaks were detected in positive mode than in negative mode. According to the well-known phytochemical compounds from Indigo Naturalis, chemical name, MS^1^ data, fragment of MS^2^ data, retention times and *m/z* values of the molecular ions, 19 chemicals were identified from INAE as Table 1 and Fig. 1: 13 alkaloids, 3 nucleosides, 1 terpene, 1 amino acid and 1 original acid.

**Table 1:** Constituents identified from INAE

Peak no	Name (possible chemical)	Categories	Formula	MS^1^	MS^2^
1	Adenine	Nucleosides	C5H5N5	136.13	119.00
2	2-Methyquinazlin-4(3H)-One	Alkaloids	C9H8N2O	161.14	140.84,133.20
3	Imidazolinones	Alkaloids	C8H7N3O	162.15	145.00
4	3-(2′-Hydroxyphenyl)-2-Methyl-4(3H)-Quinazolinone	Alkaloids	C15H12N2O2	253.16	134.09,120.06
5	Isaindigotone2	Alkaloids	C20H18N2O4	351.28	333.21,315.21
6	Adenosine	Nucleosides	C10H13N5O4	268.17	136.06
7	Annuionone D	Terpene	C13H20O3	225.18	156.28
8	6-Hydroxy-4-(5-Hydroxymethylfuran-2-yl)-Quinolin-2(1H)-One	Alkaloids	C16H19NO2	258.19	240.16
9	Valine	Amino acids	C15H11NO2	118.16	101.12,98.14
10	Hydroxylindirubin	Alkaloids	C16H10N2O3	279.31	261.25,223.18
11	Isaindigodione	Alkaloids	C18H18N2O4	349.38	331.28
12	Mediroresinol	Alkaloids	C21H24O7	349.35	371.25
13	2-(3-Hydroxy)-4-Methoxy-2-Oxindolin-3-yl)-Acetamide	Alkaloids	C11H13N2O4	237.21	118.08
14	Ganine	Nucleosides	C5H5N5O	174.17	157.13
15	Quinazolinones	Alkaloids	–	316.22	298.19,288.48
16	Imidazolinones	Alkaloids	–	354.31	337.18,175.11
17	2-Hydroxy-1,4-Phthalate	Organic Acids	C8H6O5	183.16	163.06,139.12
18	Quinazolinones	Alkaloids	–	353.22	319.25,335.22
19	Ethyl-3,4-Dihydro-4-Oxoquinazoline-2-Carboxylate	Alkaloids	C11H10N2O3	460.33	437.28,415.26

The phytochemical constituents in aqueous extract of Indigo Naturalis (INAE) were identified through UPLC-Q-TOF–MS and UPLC-LTQ-ESI–MS readouts including the retention time, MS/MS fragments, molecular form with referred to the databases SciFinder, PubChem, and Mass Bank

### Anti-influenza and anti-inflammatory effects of INAE in vitro

**Fig. 2:**
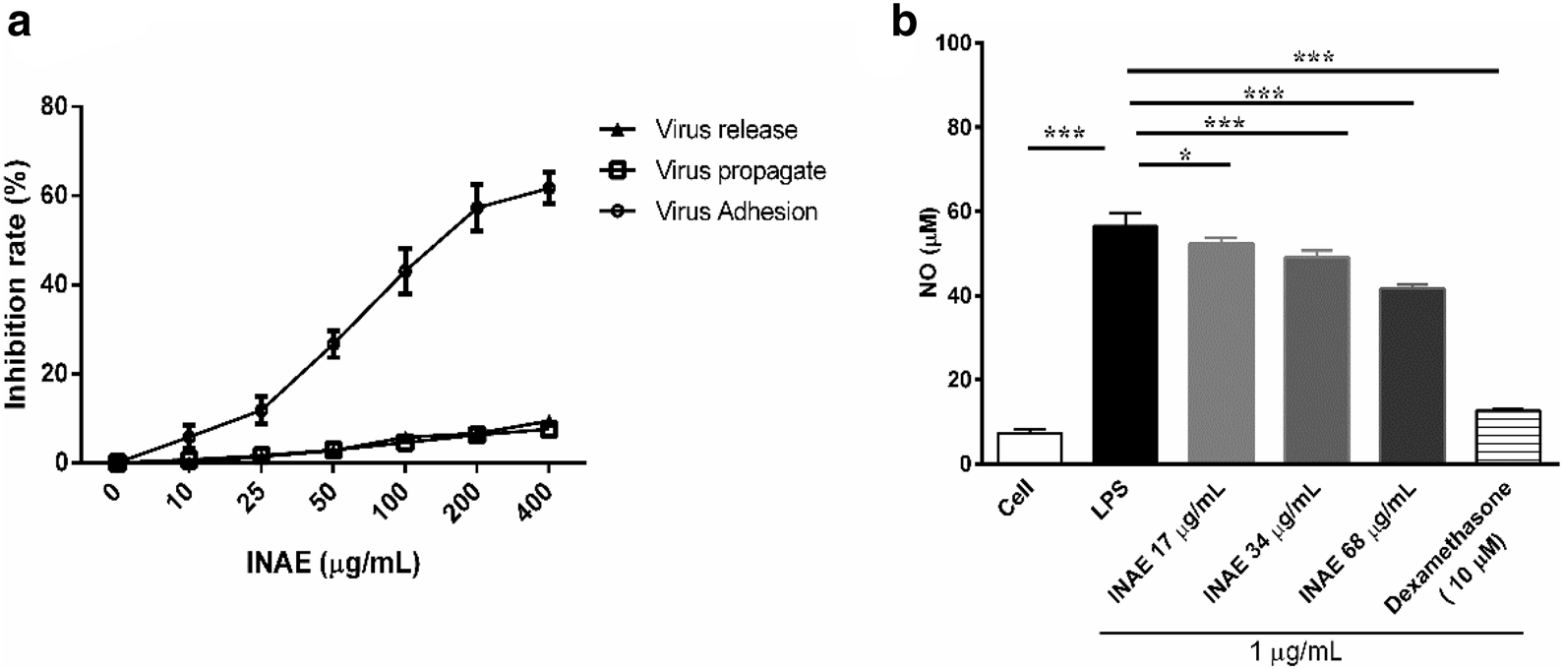
Anti-influenza and anti-inflammatory effect of INAE in vitro*.* **a** Anti-influenza effect of INAE on MDCK cells. INAE was administrated 2 h prior to, simultaneously, or 2 h after influenza A virus infection to evaluate the antivirus capacity in phases of virus adhesion to cells, virus propagate in or release from the infected cells. **b** Effect of INAE on LPS induced nitric oxide (NO) production. LPS (1 μg/mL) was used as initiators in peritoneal macrophages and dexamethasone (10 μM) was used as positive control. Data were presented as mean ± S.D. (*n* = 4). *P < 0.05, ***P < 0.001 compared with LPS group, tested by ANOVA and Fisher’s PLSD

The result showed that INAE had no toxicity on MDCK cells up to concentration of 1000 μg/mL (data not shown). As shown in Fig. 2a, INAE was administrated 2 h prior to virus infection (to interfere the adhesion process of virus to cells), the inhibition rate of virus replication gradually increased up to 60% as compound concentration increased to 400 μg/mL. No obvious inhibition was observed on the cells treated with virus mix-incubated INAE simultaneously (direct killing effect of INAE to virus) or with INAE 2 h after virus infection (to interfere the release of virion from cells). The result demonstrated that INAE inhibited the process of adhesion of virus to cells, but had no direct killing effect to virus in vitro.

As shown in Fig. 2b, compared with cell control group, LPS stimulation significantly increased NO production in peritoneal macrophages. INAE administration (17, 34 and 68 μg/mL) remarkable suppressed the elevated NO production induced by LPS (*P* < 0.05).

### INAE improved lifespan and survival rate of IAV-infected mice

**Fig. 3:**
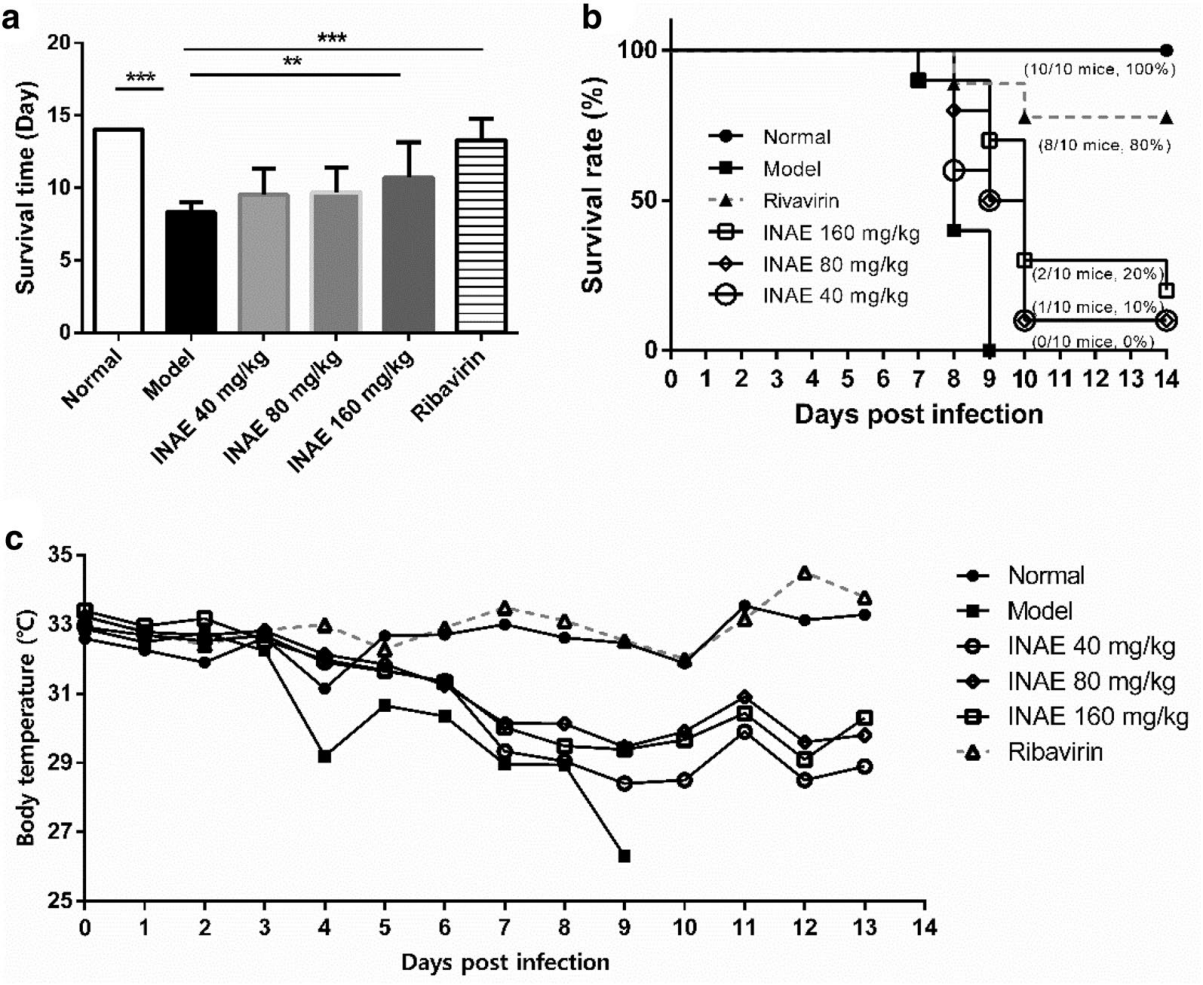
Effect of INAE on lifespan and survival rate of IAV-infected mice. Mice were intranasally infected with 6 × LD_50_ of IAV and administered orally with INAE, ribavirin or 0.5% CMC-Na at indicated doses once daily for 7 days. Mice lifespan (**a**), survival rate (**b**), and mean body temperature during the study (C) were recorded. Data represent were expressed as mean ± S.D. (*n* = 10). **P < 0.01, ***P < 0.001 compared with model group, tested by ANOVA and Fisher’s PLSD

The mice infected with IAV were observed for 14 days. The mice showed clinical signs of piloerection, ruffled fur, lack of food consumption, and body weight loss after virus inoculation. INAE significantly expanded the lifespan of IAV-infected mice (Fig. 3a). Model group mice had a notable low survival time (8.30 ± 0.67 days) compared with INAE groups mice (ranged from 9.80 ± 2.12 days to 10.80 ± 2.66 days).

There was no death in normal group during 14-day’s observation. Mice in model group were found dead since day 7 after IAV infection and 100% of the model mice died within 9 days (Fig. 3b). The survival rate of mice in model group was 0% since day 9. Mice treated with INAE (40, 80 and 160 mg/kg) were protected from lethality. When compared with model group, the survival rate of mice significantly raised in INAE 40 and 80 mg/kg groups during days 8–9 (*P* < 0.05), and in INAE 160 mg/kg group during days 8–12 (*P* < 0.05). The survival rate of INAE (40, 80 and 160 mg/kg) on day 14 were 10%, 10%, and 20%.

Animal body temperature was also reported during the study. It is worth noting that the body temperature of mice in model group was notably lower than normal group, while INAE treatment groups exhibited higher temperature than model group during the study (Fig. 3c).

### INAE alleviated IAV-induced acute lung injury

**Fig. 4:**
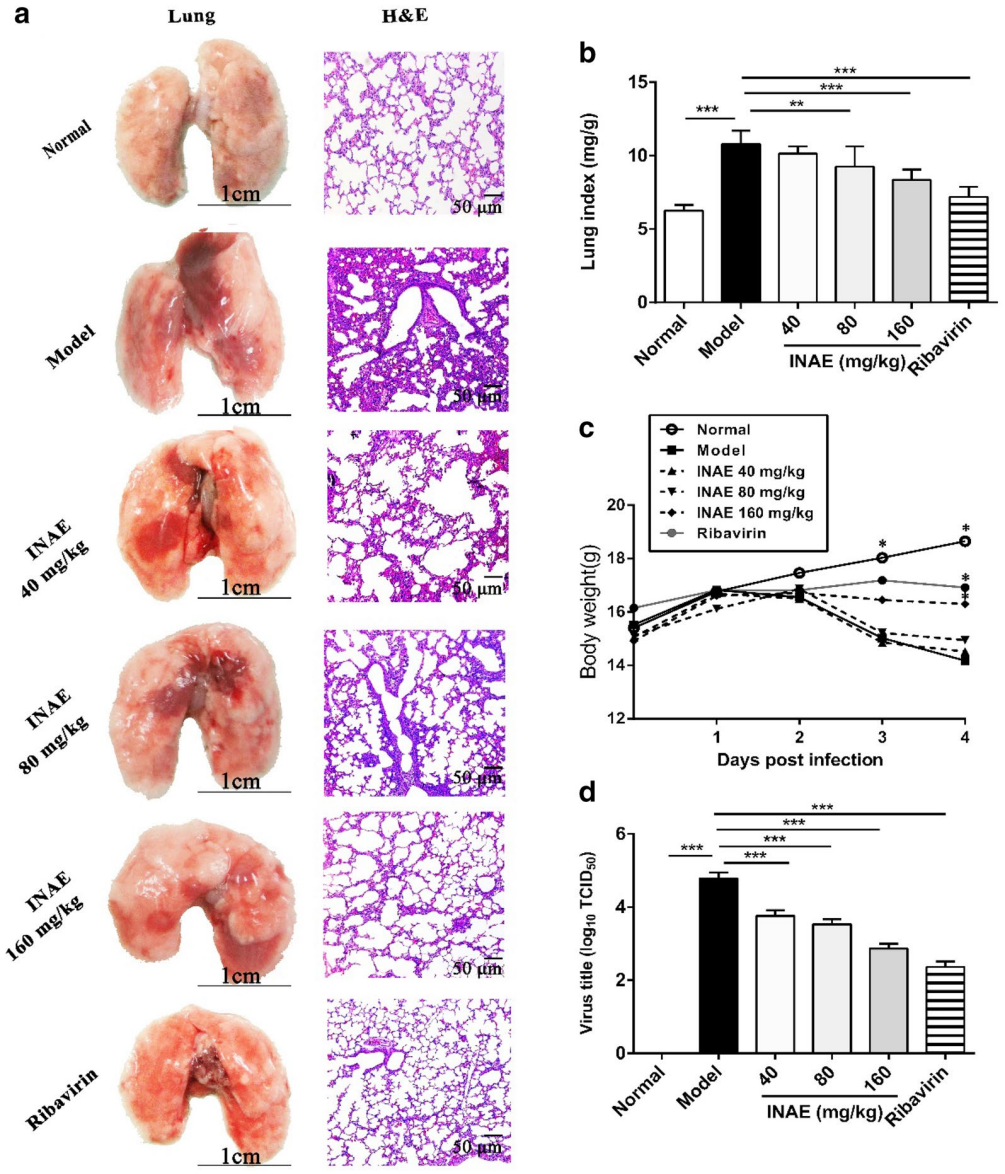
Effect of INAE on lung injury, lung index, body weight and virus titer of IAV-infected mice. Mice were infected with 3 × LD_50_ of IAV and administered orally with INAE, ribavirin or 0.5% CMC-Na at indicated doses once daily for 4 days. Lung index, mice body weight, and virus titer were calculated and detected. Lung photos and hematoxylin–eosin stain (H&E) with vision of ×200  (**a**). Lung index = Lung weight / body weight × 100% (**b**). Mice body weight growth curve (**c**). Virus titer in lung homogenate of mice (**d**). Data were presented as mean ± S.D. (*n* = 6). **P < 0.01, ***P < 0.001 compared with model group, tested by ANOVA and Fisher’s PLSD

INAE was administrated to evaluate the effect on IAV-induced ALI. Significant body weight loss and lung index increase were exhibited in mice infected with IAV. As shown in Fig. 4c, IAV infected mice began to lose body weight since day 2 after IAV infection. Compared with model group, body weight of mice treated with INAE and ribavirin were significantly higher on day 3 and day 4 (*P* < 0.05). Compared with normal group (6.26 ± 0.36 mg/g), remarkable increase of lung index was observed in model group (10.78 ± 0.93 mg/g). Comparatively, treatment with INAE (Fig. 4b) significant decreased the lung index (80 mg/kg, 9.25 ± 1.39 mg/g; 160 mg/kg, 8.34 ± 0.72 mg/g).

Pathologic findings indicated that normal group mice had lungs in terms of nature size, color, and texture with clear and intact round alveolar cells (Fig. 4a). In the contrary, large areas of alveolar cells and bronchioles were damaged and most alveolar walls were destroyed in model group. Amounts of inflammatory cells infiltration was observed in injured alveolus and pneumocytes drop off from bronchiole. Histological damages in lung of mice were obviously alleviated in INAE treated groups. Inflammatory cell infiltration was significantly decreased compared with model group.

### INAE lowered virus titer in lung of IAV-infected mice

Acute lung injury referred to virus replication and virus titer were determined by TCID_50_. As shown in Fig. 4d, virus titer in lung homogenate of mice treated with INAE (40 mg/kg, 3.78 ± 0.15; 80 mg/kg, 3.50 ± 0.20; 160 mg/kg, 2.87 ± 0.12) and ribavirin (2.36 ± 0.14) were clearly lower than that of model group (4.78 ± 0.16) on day 4 after IAV infection (*P* < 0.05). The result suggested that the decrease of virus titer was accordance with the protective effect of INAE on acute lung injury.

### INAE reduced injury in liver, thymus and spleen

**Fig. 5:**
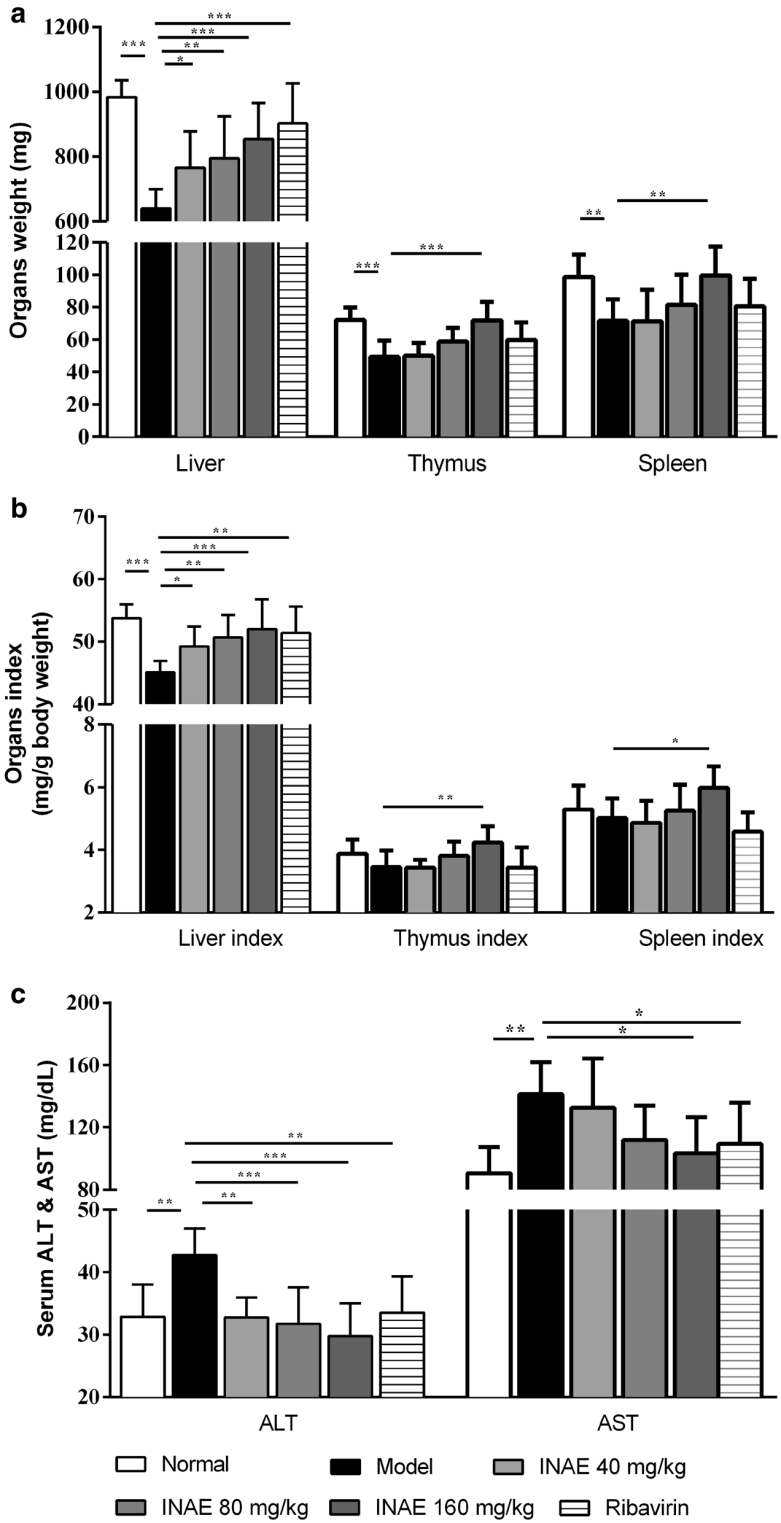
Effect of INAE on liver, thymus and spleen. Mice were infected with 3 × LD_50_ of IAV and administered orally with INAE, ribavirin or 0.5% CMC-Na at indicated doses once daily for 4 days. Weights of liver, thymus and spleen were measured and calculated (**a**, **b**). Serum ALT and AST levels were evaluated (**c**). Organ index = Organ weight / body weight × 100%. Data were presented as mean ± S.D. (*n* = 6). ^*^ *P* < 0.05, **P < 0.01, ***P < 0.001 compared with model group, tested by ANOVA and Fisher’s PLSD

As shown in Fig. 5a and b, weights of liver, thymus and spleen of mice in model group were significantly decreased compared with normal group (*P* < 0.05). Comparatively, INAE administration (40, 80 and 160 mg/kg) significant increased weights and organ index of liver, and administration of 160 mg/kg of INAE significantly increased both weight, organ indexes of thymus and spleen.

Serum ALT and AST are the main predictor of liver injury [31]. As shown in Fig. 5c, serum ALT and AST levels increased significantly in model group compared with normal group (*P* < 0.05). INAE administration significantly decreased ALT (40, 80 and 160 mg/kg) and AST (160 mg/kg) levels compared with model group (*P* < 0.05).

### INAE suppressed the expression of HMGB-1 and TLR4 in lung of IAV-infected mice

**Fig. 6:**
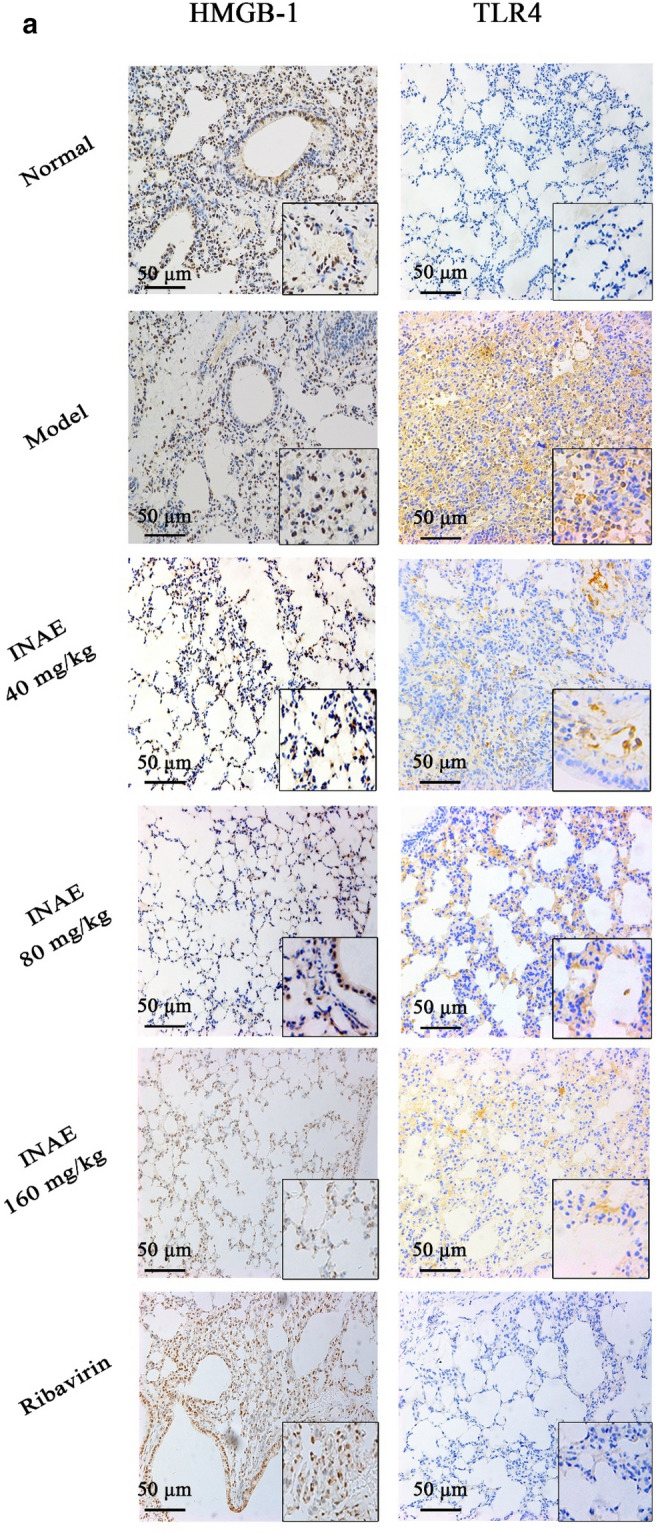

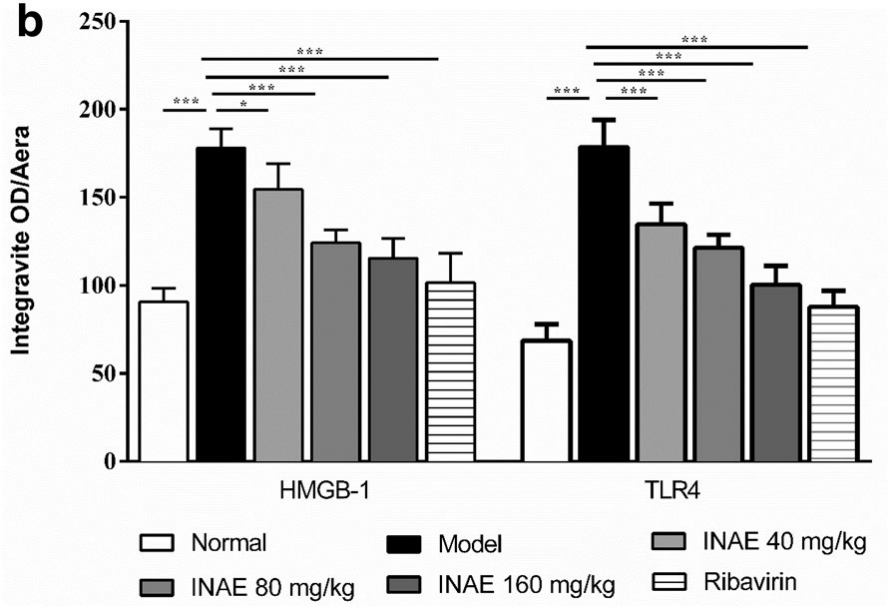
Expression of HMGB-1 and TLR4 in lung of IAV-infected mice. Lung tissues of IAV-infected mice were harvested, fixed in formalin, and embedded in paraffin and processed for slices preparation. **a** The slides were incubated with relative antibodies against HMGB-1 and TLR4, stained with chromogenic substrate solution and counterstained with hematoxylin. The immuno-histochemical (×200) of expression of HMGB-1 and TLR4 in lung of IAV-infected mice were visualized under a microscope. **b** The expression of HMGB-1 and TLR4 were assessed by semi-quantitative analysis using ImageJ software. The levels of HMGB-1 or TLR4 were expressed as average optical value (AOD). AOD = Integrative density of optical value / Aera. Data were expressed as mean ± S.D. (*n* = 4). *P < 0.05, ***P < 0.001 compared with model group, tested by ANOVA and Fisher’s PLSD

As shown in Fig. 6a, mice in normal group basically expressed HMGB-1 in nuclei of lung tissue. Compared with normal group, the expression of HMGB-1 in model group increased and a large amount of HMGB-1 was found out of nuclei and cells. INAE and ribavirin reduced the over expression and the release of HMGB-1. Mice in normal group expressed a small quantity TLR4, while the expression increased in model group. Immunohistochemistry staining of HMGB-1 and TLR4 were also assessed by semi-quantitative analysis using ImageJ software (Fig. 6b). Compared with model group, the expression of HMGB-1 and TLR4 were significantly decreased (*P* < 0.05) as the concentration of compound increased.

### INAE increased anti-oxidant capacity and inhibited oxidative stress

**Fig. 7:**
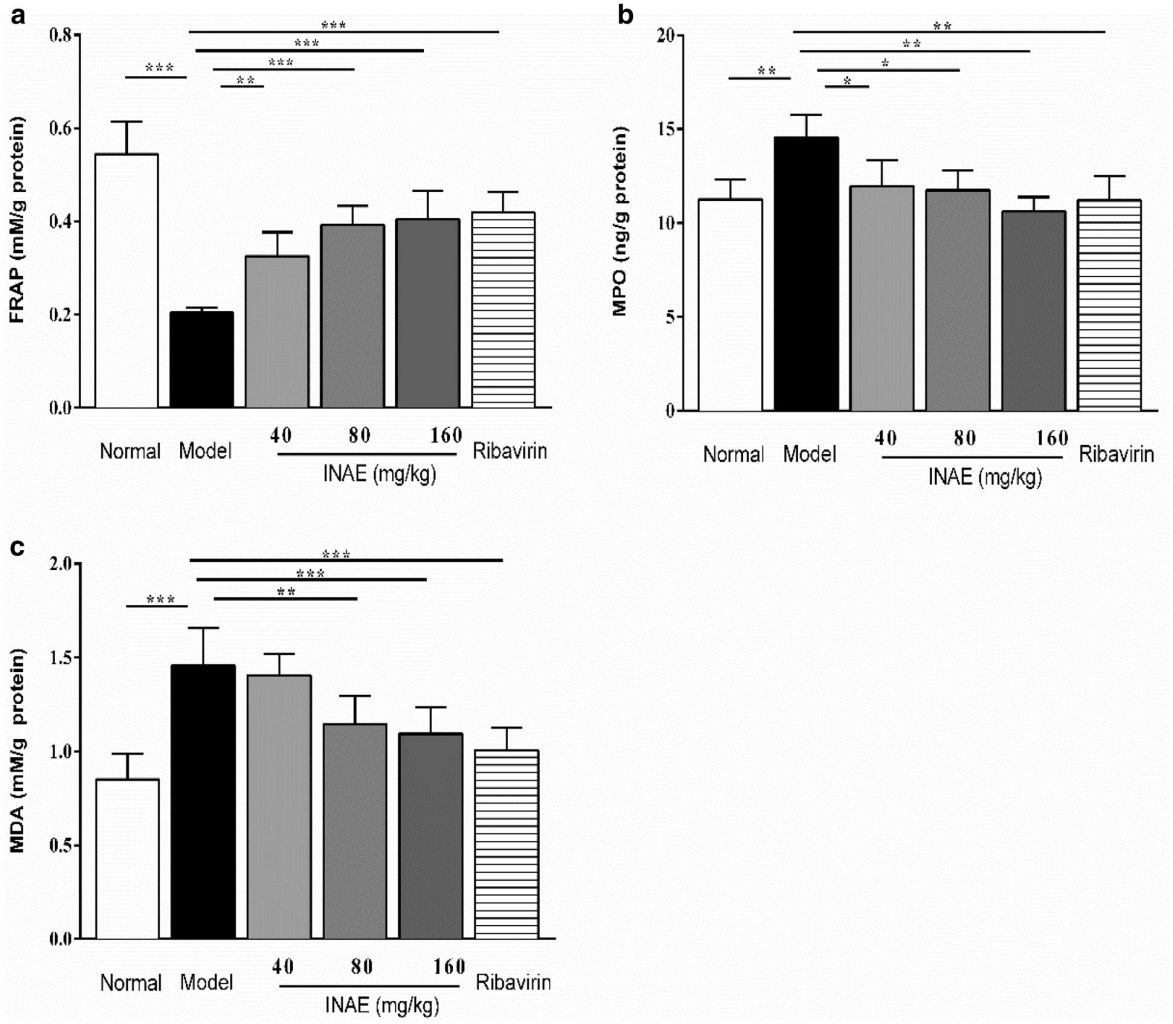
Anti-oxidant effect of INAE in lung of IAV-infected mice. **a** Ferric ion reducing antioxidant power (FRAP), **b** Myeloperoxidase (MPO) and **c** Malonaldehyde (MDA) in lung homogenate of IAV-infected mice were analyzed to evaluate the effect of INAE on oxidant stress reaction. Data were expressed as mean ± S.D. (*n* = 6). *P < 0.05, **P < 0.01, ***P < 0.001 compared with model group, tested by ANOVA and Fisher’s PLSD

As shown in Fig. 7, the FARP value in lung homogenate of mice in model group significantly decreased compared with that in normal group (*P* < 0.05). INAE administration significantly increased the FARP value compared with model group (*P* < 0.05). The levels of MPO and MDA in lung homogenate were significantly increased in model group compared with normal group (*P* < 0.05). In comparison with model group, INAE administration markedly reduced production of MPO and MDA (*P* < 0.05). The decrease of production of MPO and MDA demonstrated the remarkable anti-oxidant capacity of INAE.

### INAE inhibited inflammatory response in lung of IAV-infected mice

**Fig. 8:**
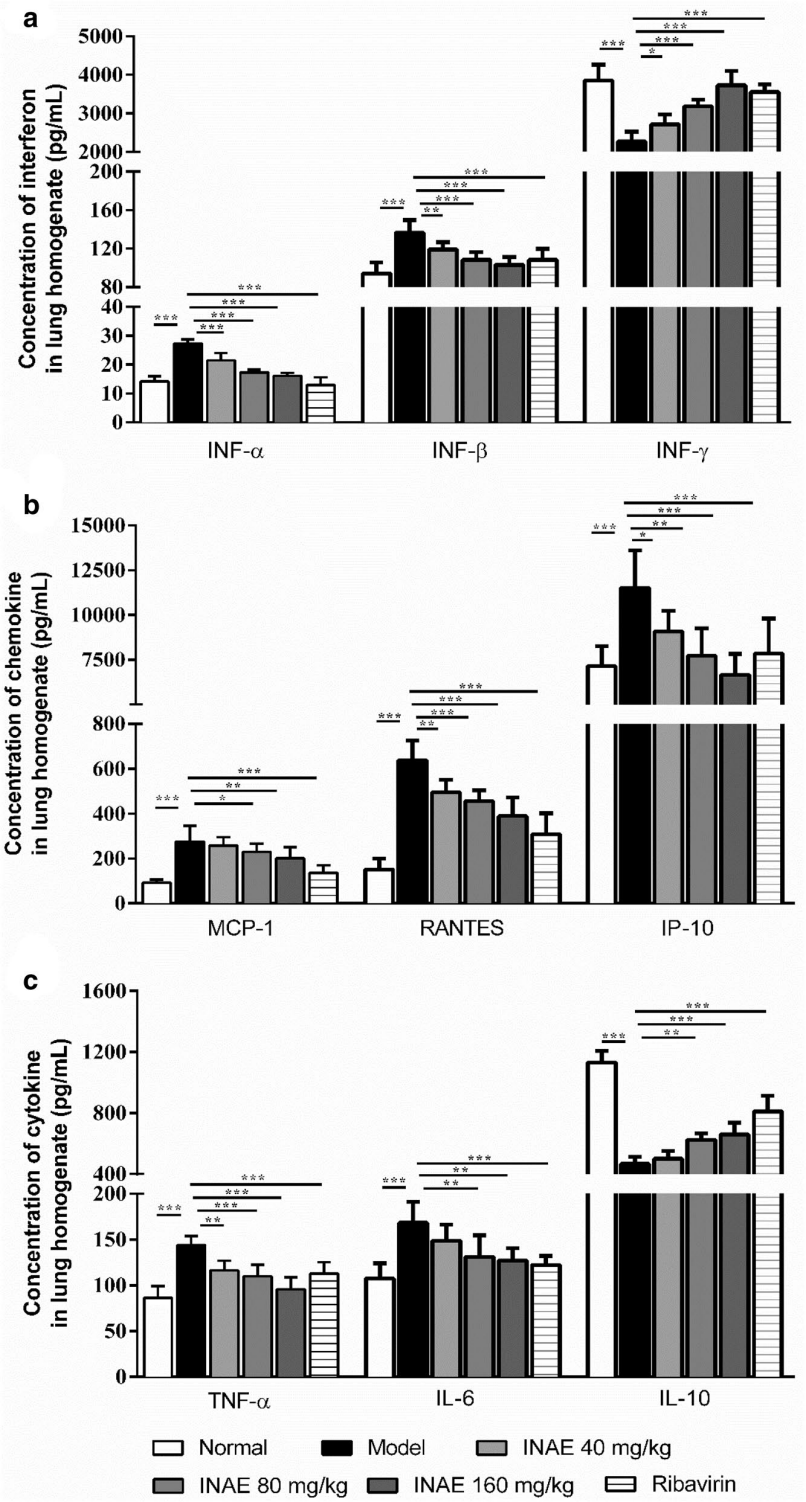
Interferon, chemokine and cytokine levels in lung of IAV-infected mice**.** Effect of INAE on inflammation response in IAV-infected mice were detected at day 4 after IAV infection. Interferon IFN-α, IFN-β, and INF-γ (**a**), MCP-1, RANTES and IP-10 (**b**), and TNF-α, IL-6, and IL-10 (**c**) levels were evaluated. Data were expressed as mean ± S.D. (*n* = 6). *P < 0.05, **P < 0.01, ***P < 0.001 compared with model group, tested by ANOVA and Fisher’s PLSD

As shown in Fig. 8, the levels of IFN-α, IFN-β in lung homogenate significantly increased in model group compared with normal group (*P* < 0.05). In comparison with model group, INAE administration significantly reduced IFN-α and IFN-β expression (*P* < 0.05). The level of IFN-γ significantly decreased in model group and INAE administration significantly increased IFN-γ expression (*P* < 0.05).

In comparison with normal group, model group exhibited significant increase in levels of chemokines MCP-1, RANTES and IP-10 (*P* < 0.05). Treatment with INAE significantly decrease the expression of MCP-1, RANTES and IP-10 (*P* < 0.05) in lung homogenate of IAV-infected mice.

Moreover, the expression of TNF-α and IL-6 in lung homogenate of mice in model group significantly increased compared with normal group (*P* < 0.05). INAE administration markedly reduced the expression of TNF-α and IL-6 (*P* < 0.05). IL-10 is an important anti-inflammatory factor that can ameliorate immunopathology by limiting immune responses involved in tissue damage. The expression of IL-10 significantly decreased in lung homogenate of mice in model group compared with normal group (*P* < 0.05). The administration of INAE significantly increased IL-10 production in lung homogenate of IAV-infected mice (*P* < 0.05).

## Discussion

Traditional Chinese medicine is effective in prevention and enhancing the resistance to pandemic with unique insights [32]. Many traditional Chinese herbs, for instance, *Radix isatidis* (Banlangen), *Houttuynia cordata* (Yuxingcao), *Radix Scutellariae* (Huangqin)*, Folium isatidis* (Daqingye) were proved to exhibit anti-virus effect during pandemics of SARS in 2003 [33–35]. During the treatment period of COVID-19, TCM scheme was included in the guideline on diagnosis and treatment of COVID-19, and TCM fully participate in the whole rescue process [36]. Decoction is the main form of TCM [37]. Yin-Ku Lin et al*.* found that the abstracts of Indigo Naturalis in oil reduced nail psoriasis severity index in patient, and the abstracts of Indigo Naturalis in dimethyl sulfoxide suppressed the increase of protein carbonyl groups in human keratinocytes [38, 39]. However, as the main therapeutic dosage form of TCM, there are few researches regarding the effect of Indigo Naturalis on IAV-induced ALI. In our study, INAE as the aqueous extract of Indigo Naturalis was studied referred to clinical use of TCM.

After herbal extract preparation, the constituents of INAE was analyzed using an UPLC-ESI-LTQ-MS system. In the experiment, more plentiful chromatographic peaks were detected in positive mode than in negative mode, and positive mode was chosen to characterize the chemical constituents of INAE. According to the retention times and *m/z* values of the molecular ions, 19 chemicals, including 13 alkaloids, 3 nucleosides, 1 amino acid, 1 terpene and 1 organic acid, were identified through the comparison with the standard compounds and the database of known chemicals. Among these chemicals, alkaloids were the main small molecule in Indigo Naturalis [40, 41]. Alkaloids has been proven to exhibit antiviral efficacy and inhibit LPS induced inflammation [42, 43].

The process of virus infection to host cells has three different phases: attachment of virus to host cells, virus replication in and release of virion from host cells [24]. The anti-influenza effect of INAE was evaluated in vitro first. INAE were inoculated 2 h prior to, simultaneously, or 2 h after IAV infection with MDCK cells to test the ani-virus efficacy in three different phases. In vitro results shown that INAE markedly inhibited virus adhesion to cells when administrated before virus infection, but had no inhibitive effect on virus replication and release.

In our study, INAE suppressed the elevated NO production from LPS-stimulated peritoneal macrophages in vivo*.* NO produced by inducible nitric oxide synthetase (iNOS) is an important proinflammatory mediator. NO has direct antiviral properties against some viruses, whereas during virus infections NO can mediate immunopathology and/or inhibit the antiviral immune response to promote chronic infection [44]. Accumulated evidence suggests that NO and oxygen radicals such as superoxide are key molecules in the pathogenesis of various infectious diseases and accelerate tissue damage [45]. Free radical such as NO is highly active chemical substances which is exceptionally destructive, indiscriminately causing protein deterioration, cell membrane destruction, DNA damage, cell death, and organ failure [46]. It is known that virus infection induces massive production of free radicals [47]. Most cytokines, such as IFN-γ, IL-1β, IL-2, IL-6, TNFα can all stimulate the generation of NO [48]. Inhibition of NO synthesis can decrease the production of IL-6 and the cytotoxicity effect of inflammatory cytokines can be blocked by lipid peroxidation inhibitor [49]. Mice deficient in inducible NO synthase exhibited reduced morbidity and mortality when challenged with influenza virus, which told us that NO is the culprits in the virus induced pneumonia death [50]. The in vitro effect of INAE on inhibiting the over production of NO might be helpful for reducing the production of inflammatory cytokines and oxidative tissue damage in IAV-infected mice.

Our study showed that treatment with INAE significantly expanded the lifespan and increased the survival rate of IAV-infected mice. Oral administration of INAE significant alleviated lung injury with reduced lung index and virus titer, as well as decreased inflammatory cells infiltration in lung. The results that INAE can alleviate acute lung injury is accordance with clinic treatment [20].

The model of acute lung injury induced by IAV is usually used for research of anti-influenza agents. Once mice were infected with IAV, amounts of influenza virus infecting pneumocytes results in cells necrosis and apoptosis in lung tissues and causes acute lung injury [51]. Disorder of balance between inflammation and anti-inflammation led to death of mice for the reason of continuous injury of lung and other organs [52]. In our study, severe injury of vital organs, including lung, liver, thymus and spleen were observed in IAV-infected mice. Administration of INAE alleviated injury of vital organs, especially decreased lung weight and lung index. The liver weight and index, as well as the serum ALT and AST production were reduced by INAE treatment. Spleen and thymus injury were also released in high group of INAE administration. Like influenza virus infection, liver damage and hepatic necrosis, and splenic tissue were shown to be atrophic in the current outbreak of COVID-19 infected patient [53, 54]. Most of the recommended prescriptions consists TCM, which have the efficacy of heat-clearing and detoxicating. Indigo Naturalis also has the similar feature, which provide a possibility that INAE might make contribution to attenuate multi organ failure in COVID-19 and more researches are needed for further discovery [55, 56].

HMGB-1, a nuclear non-histone protein that is released or secreted from the cells in response to infections or damage, is a sentinel for the immune system that plays a critical role in cell survival/death pathways [57]. HMGB-1 is considered as an essential facilitator in diseases such as acute lung injury and local and systemic inflammation [58]. HMGB-1 was implicated in host tissue destruction and persistent pathological changes in IAV-infected hosts [59]. TLR4, one of the important inflammatory signal receptors of the pathogen recognition receptors family (PRR), plays a critical role in the activation of immune system as influenza virus and COVID-19 invasion [60, 61]. TLR4 is also an important transmembrane protein for the development of cytokines production [62]. There is growing evidence that TLR4 signal inhibition alleviated lung injury in IVA infected mice [63, 64]. Modulation of HMGB1/TLR4 signaling pathway provides a way in the management of ALI induced by IAV.

Lung cell damage caused by IAV infection and inflammation releases a large amount of HMGB-1 [11, 65]. As a kind of DAMP, high level of HMGB-1 expression in lung leads to continuous activation of TLR4 signaling pathway and stimulates the inflammatory cells to continuously release cytokines, chemokines, as well as inducible nitric oxide, which accumulate in the injured lung tissue and further aggravate the tissue damage [66, 67]. Large numbers of neutrophils recruit to the damaged lung tissue induced by chemokines, and secrete excessive peroxide products while participating in the immune process [50]. Virus replication in host cells, excessive production of NO and peroxidation products such as MPO released from neutrophils recruited to the damaged tissues contribute to the exacerbation of inflammation and lung injury [68]. Our experiment demonstrated that INAE inhibited over expression of HMGB-1 and TLR4 that might benefit the alleviation of lung injury in IAV infected mice.

Some cases showed that neutrophil infiltration contributed to cytokine storm in virus infection [53]. FRAP is a global indicator of antioxidant capacity [69]. MPO is an enzyme mainly found in azurophilic granules of neutrophils, which serves as a good marker of inflammation, tissue injury and neutrophil infiltration [70]. Oxidative damage may represent crucial pathogenic factor in acute lung injury due to the increased production of reactive oxygen and nitrogen species [71]. MDA is the main products of lipid peroxidation and mediates inflammation [72]. Treatment with INAE increased FRAP, and decreased MPO, MDA production in lung homogenate of IAV-infected mice, which demonstrated that INAE increased anti-oxidant capacity and suppressed oxidant stress.

Excessive production of proinflammatory factors plays a critical role in the pathogenesis of influenza virus infection. Lung damage and clinic symptoms associated with aberrant and uncontrolled cytokine production could ultimately lead to death [73]. TNF-α and IL-6 were the early immune cytokines contribute to the proinflammatory production [74]. Chemokines then give the signal for the directed migration of neutrophils or macrophages into the tissues. MCP-1 and RANTES are the main chemokines during early infection stage of influenza [8, 75]. IP-10 belongs to the chemokine CXC subfamily and acts as a chemo attractor for T cells and NK cells. IL-10 is a critical protective modulator which can attenuate the activation of lymphocytes and inflammatory cascades during virus infection. Importantly, a research demonstrated that induction of IP-10 in lung attracted pulmonary neutrophils, and led to lung inflammation during influenza infection [76]. Increasing IP-10 induction was consistently found in the serum of H5N1-infected patients and animal models [77]. The present study demonstrated that treatment with INAE remarkable modulated levels of antivirus factors (IFN-α, IFN-β and IFN-γ), decreased levels of chemokines MCP-1, RANTES and IP-10, and reduced the production of proinflammatory factors TNF-α and IL-6. Meanwhile, INAE significantly increased the production of anti-inflammatory factor (IL-10). It is all known that the aggressive pro-inflammatory response and insufficient control of anti-inflammatory response in virus infection such as influenza infection, SARS and COVID-19, lead to systemic cytokine storm and accelerate tissue injury [53]. Our study demonstrated the inhibition effects of INAE on cytokines and chemokines, peroxide production, and suppression of neutrophilia infiltration (reflected by MPO level in Fig. 7). Based on the beneficial effect of INAE, we supposed that INAE might reduce the risk of cytokine storm in severe virus infection.

Indigo Naturalis mainly contains indigo, indirubin, tryptanthrin and other alkaloids, as well as indigo ketone, terpenoids, sitosterol and amino acids [78, 79]. Indigo and indirubin are the main components of Indigo Naturalis and are the two important indicators for the quality of Indigo Naturalis in the Chinese Pharmacopoeia [17], and indirubin is generally taken as the major effective constituents of Indigo Naturalis for the treatment of acute promyelocytic leukemia and inflammation and autoimmune diseases such as colitis and psoriasis [21, 80, 81]. However, we found that neither indigo nor indirubin could alleviate IAV-induced ALI by oral administration (data were shown in Additional file 1: Fig. S1). In order to reveal the potential effective components of INAE, 19 compounds were recognized from INAE by the method of LC–MS/MS, among which 13 components were alkaloids. We supposed that one or several other kinds of alkaloids different from indigo and indirubin might be the active ingredients that contributed to the beneficial effect of INAE. We will isolate these potential active alkaloids from INAE and evaluate their in vivo effects on ALI in our future research.

Our research studied the anti-inflammatory and antiviral effects of Indigo Naturalis both in vivo and in vitro. INAE inhibited the adhesion process of virus to host cell in vitro to reduced virus replication, but had no direct killing effect on virus. In vivo experiment showed INAE treatment extended the lifespan and reduced the mortality of IAV-infected mice, and reduced virus titer in lung tissue, which demonstrated the antiviral effect of INAE in vivo. On the other hand, INAE inhibited the production of NO induced by LPS, showing an anti-inflammatory effect in vitro. In vivo experiments showed that INAE inhibited the HMGB-1 and TLR4 signaling pathway, reduced the extensive production of pro-inflammatory cytokines and chemokines, as well as the tissue-damaging peroxide products. Our present study showed that INAE effectively alleviated ALI caused by IAV infection, and the underlying action mechanisms may be closely associated with the anti-influenza, anti-inflammatory and anti-oxidant activities of INAE. The inhibition effect of INAE on HMGB-1/TLR4 signaling pathway might contribute to improve the lung injury induced by the inflammation and oxidative stress.

## Conclusion

**Fig. 9:**
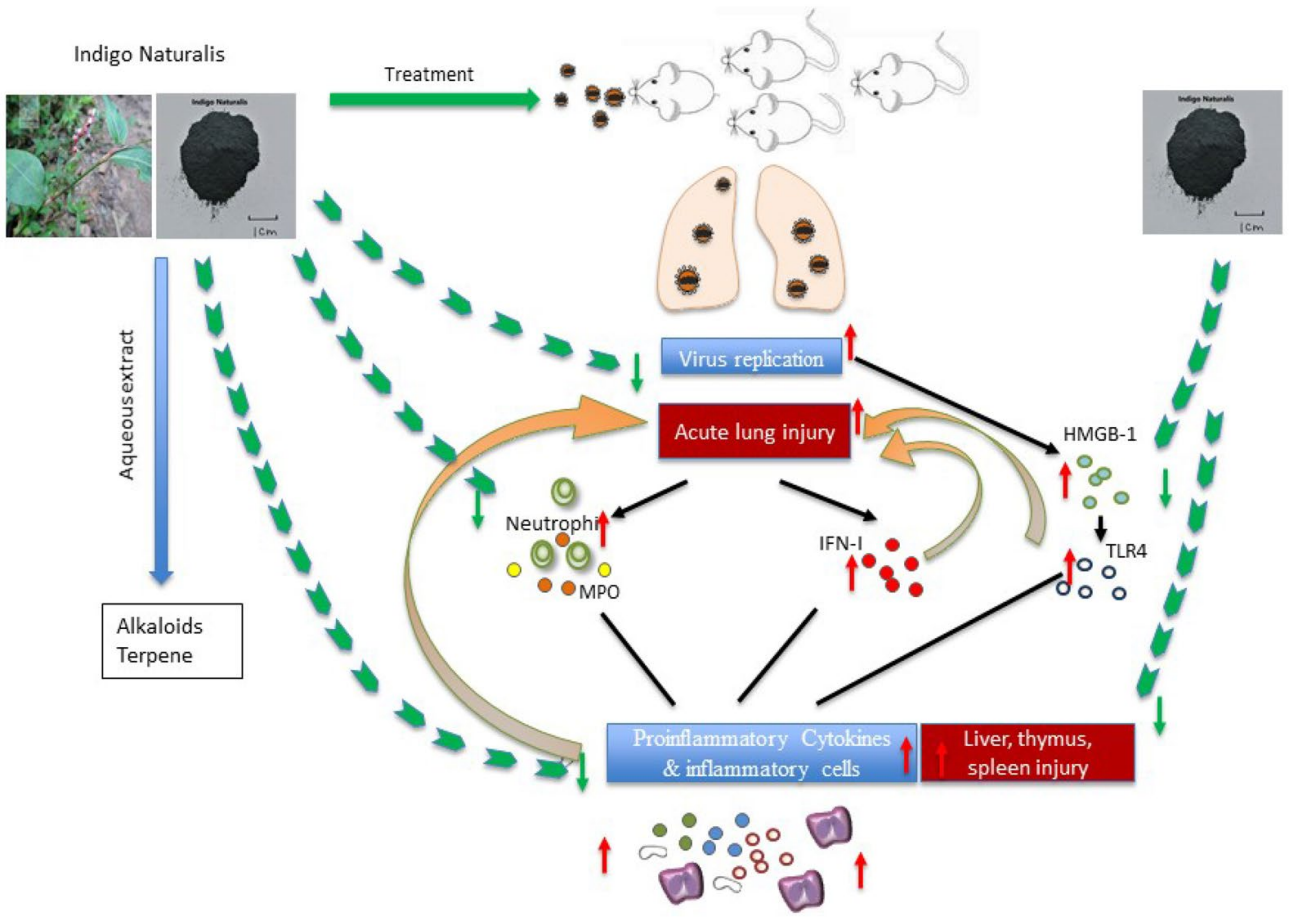
Graphic abstract of IAV-induced acute lung injury. Influenza A virus infected host alveolar epithelial cells and caused necrosis and injury in lung. The innate immune response began with the virus recognition and was amplified through activation of HMGB-1 and TLR4 signaling pathway. Extensive production of cytokines, chemokines produced by inflammation cells and amounts of peroxide products released from neutrophils exacerbated the injury of lung and multi organ failure in liver, thymus and spleen (red arrow). INAE treatment alleviated the acute lung injury and multi organ damage (green arrow) through the effects of anti-virus, anti-inflammatory and anti-oxidation

In summary, our investigation described the beneficial effect of Indigo Naturalis against ALI in IAV infected mice, and the underlying mechanism might be closely associated with its inhibition of virus replication, anti-inflammatory and anti-oxidant effects (Fig. 9). As acute lung injury, multi organ failure such as liver, thymus and spleen, and cytokine storm occurred both in influenza virus and SARS-CoV-2 infection, our study suggested Indigo Naturalis could be a promising agent and warrant further evaluation for treatment of influenza A virus induced acute lung injury and SARS-CoV-2 induced COVID-19.

## Supplementary Information

**Additional file 1: Figure S1**. Effect of indigo and indirubin on ALI in IAV-infected mice. Mice were infected with 3 × LD_50_ of IAV and then administered orally with indigo (6 mg/kg, 30 mg/kg), indirubin (6 mg/kg, 30 mg/kg), ribavirin (100 mg/kg) or 0.5% CMC-Na once daily for 4 days. The mice were sacrificed on day 4 after IAV infection. Mice body weight and lung index were reported and calculated. (A) Lung index = Lung weight / body weight × 100%. (B) Mice body weight growth curve. Data were presented as mean ± S.D. (*n* = 6 ~ 8). *P < 0.05, ***P < 0.001 compared with model group, tested by ANOVA and Fisher’s PLSD.

## Data Availability

The datasets used and/or analyzed during the current study are available from the corresponding author on reasonable request.

## Abbreviations

INAE: Aqueous extract of Indigo Naturalis
ALI: Acute lung injury
IAV: Influenza A virus
COVID-19: Coronavirus disease 19
SARS-CoV-2: Severe acute respiratory coronavirus 2
PAMP: Pathogen-associated molecular pattern
DAMP: Damage-associated molecular pattern
PRR: Pathogen recognition receptors
HMGB-1: High mobility group box-1 protein
TLR4: Toll-like receptor 4
TCM: Traditional Chinese medicine
LD_50_: 50% of lethal dose
MDCK: Madin-Darby canine kidney
DMEM: Dulbecco’s modified Eagle’s medium
MTT: 3-(4,5-Dimethyl-2-thiazolyl)-2,5-diphenyl-2-H-tetrazolium bromide
OD: Optical density
CMC-Na: Carboxymethyl cellulose sodium
DAB: Diaminobezidin
IFN-α: Interferon α
IFN-β: Interferon β
IFN-γ: Interferon γ
MCP-1: Monocyte chemoattractant protein-1
RANTES: Regulated upon activation normal T cell expressed and secreted factor
IP-10: Interferon induced protein-10
TNF-α: Tumor necrosis factor-α
IL-6: Interleukin-6
IL-10: Interleukin-10
FRAP: Ferric ion reducing antioxidant power
MPO: Myeloperoxidase
MDA: Malonaldehyde
NO: Nitrogen oxide
LPS: Lipopolysaccharide
H&E: Hematoxylin and eosin
ANOVA: One-way analysis of variance
S.D.: Standard deviation
