# Supplementary material for: Beneficial effect of Indigo Naturalis on acute lung injury induced by influenza A virus
Source: Chin Med. 2020 Dec 21;15:128. doi: 10.1186/s13020-020-00415-w (PMC7750395; doi:10.1186/s13020-020-00415-w)
Supplement: Supplementary file 1 — Additional file 1: Figure S1. Effect of indigo and indirubin on ALI in IAV-infected mice. Mice were infected with 3 × LD50 of IAV and then administered orally with indigo (6 mg/kg, 30 mg/kg), indirubin (6 mg/kg, 30 mg/kg), ribavirin (100 mg/kg) or 0.5% CMC-Na once daily for 4 days. The mice were sacrificed on day 4 after IAV infection. Mice body weight and lung index were reported and calculated. (A) Lung index = Lung weight / body weight × 100%. (B) Mice body weight growth curve. Data were presented as mean ± S.D. (n = 6 ~ 8). *P < 0.05, ***P < 0.001 compared with model group, tested by ANOVA and Fisher’s PLSD. [file 13020_2020_415_MOESM1_ESM.doc]

| **Additional file**  **Figure of Contents** | |
| --- | --- |
|  | |
| Figure S1. Effect of indigo and indirubin on ALI in IAV-infected mice |  |





**Figure S1. Effect of indigo and indirubin on ALI in IAV-infected mice**

Mice were infected with 3 × LD_50_ of IAV and then administered orally with indigo (6 mg/kg, 30 mg/kg), indirubin (6 mg/kg, 30 mg/kg), ribavirin (100 mg/kg) or 0.5% CMC-Na once daily for 4 days. The mice were sacrificed on day 4 after IAV infection. Mice body weight and lung index were reported and calculated. (A) Lung index = Lung weight / body weight × 100%. (B) Mice body weight growth curve. Data were presented as mean ± S.D. (*n* = 6 ~ 8). ^*^ *P* < 0.05, ^***^ *P* < 0.001 compared with model group, tested by ANOVA and Fisher’s PLSD.
